# Nuclear Expression of the Deubiquitinase CYLD Is Associated with Improved Survival in Human Hepatocellular Carcinoma

**DOI:** 10.1371/journal.pone.0110591

**Published:** 2014-10-16

**Authors:** Stefan Welte, Toni Urbanik, Christin Elßner, Nicole Kautz, Bruno Christian Koehler, Nina Waldburger, Justo Lorenzo Bermejo, Federico Pinna, Karl-Heinz Weiss, Peter Schemmer, Dirk Jaeger, Thomas Longerich, Kai Breuhahn, Henning Schulze-Bergkamen

**Affiliations:** 1 National Center for Tumor Diseases, Department of Medical Oncology, Internal Medicine VI, Heidelberg University Hospital, Heidelberg, Germany; 2 Institute of Pathology, Heidelberg University Hospital, Heidelberg, Germany; 3 Institute of Medical Biometry and Informatics, Heidelberg University Hospital, Heidelberg, Germany; 4 Department of Gastroenterology, Toxicology, and Infectious Diseases, Heidelberg University Hospital, Internal Medicine IV, Heidelberg, Germany; 5 Department of General and Transplant Surgery, Heidelberg University Hospital, Heidelberg, Germany; 6 Department of Gastroenterology, Diabetology and Rheumatology, Internal Medicine II, Marien-Hospital, Wesel, Germany; University of Louisville, United States of America

## Abstract

**Background & Aims:**

The deubiquitinase CYLD removes (K-63)-linked polyubiquitin chains from proteins involved in NF-κB, Wnt/ß-catenin and Bcl-3 signaling. Reduced CYLD expression has been reported in different tumor entities, including hepatocellular carcinoma (HCC). Furthermore, loss of CYLD has been shown to contribute to HCC development in *knockout* animal models. This study aimed to assess subcellular CYLD expression in tumor tissues and its prognostic significance in HCC patients undergoing liver resection or liver transplantation.

**Methods:**

Subcellular localization of CYLD was assessed by immunohistochemistry in tumor tissues of 95 HCC patients undergoing liver resection or transplantation. Positive nuclear CYLD staining was defined as an immunhistochemical (IHC) score ≥3. Positive cytoplasmic CYLD staining was defined as an IHC score ≥6. The relationship with clinicopathological parameters was investigated. Cell culture experiments were performed to analyze subcellular CYLD expression *in vitro*.

**Results:**

Cytoplasmic CYLD expression was observed in 57 out of 95 (60%) HCC specimens (^cyt^°CYLD^+^). Nuclear CYLD staining was positive in 52 out of 95 specimens (55%, ^nuc^CYLD^+^). 13 out of 52 ^nuc^CYLD^+^ patients (25%) showed a lack of cytoplasmic CYLD expression. ^nuc^CYLD^+^ was associated with prolonged overall survival in patients after resection or liver transplantation (*P* = 0.007). 5-year overall survival rates were 63% in ^nuc^CYLD^+^ *vs.* 26% in ^nuc^CYLD^-^ patients. Nuclear CYLD staining strongly correlated with tumor grading (*P*<0.001) and Ki67 positivity (*P* = 0.005). ^nuc^CYLD^+^ did not prove to be an independent prognostic parameter. *In vitro*, Huh7, Hep3B and HepG2 showed reduced CYLD levels compared to the non-malignant liver cell line THLE-2. Induction of CYLD expression by doxorubicin treatment led to increased cytoplasmic and nuclear expression of CYLD.

**Conclusions:**

Expression of nuclear CYLD is a novel prognostic factor for improved survival in patients with HCC undergoing liver resection or transplantation.

## Introduction

Hepatocellular carcinoma (HCC) is the third most common cancer-related cause of death in both sexes worldwide [1], [2]. In unresectable HCC, prognosis of patients is poor, with only limited treatment options. Thus, there is a need to identify new targets for diagnosis and treatment of the disease [3].

Ubiquitination controls the half-life of proteins, but also acts as modulator of the enzymatic activity or interaction of proteins. Ubiquitination is a dynamic process that can be counterbalanced by deubiquitinating enzymes such as Cylindromatosis (CYLD). CYLD has initially been identified as a tumor suppressor gene mutated in familial cylindromatosis, an autosomal-dominant predisposition to multiple tumors of skin appendages [4]. In addition, dysregulation of CYLD has been demonstrated in various other cancer entities [5]. CYLD removes lysine (K-63)-linked polyubiquitin chains from proteins regulating signaling pathways involved in cancer development and progression. Various deubiqitinating enzymes are predominantly localized in the nucleus and have been identified to target histones, influencing chromatin structure and downstream DNA-based processes [6]. CYLD was shown to be located in the cytoplasma and, upon stimulation, translocates to the perinuclear region where it binds to the IκB family member Bcl-3 and removes K63-linked polyubiquitin chains, thus preventing nuclear translocation of Bcl-3-p50 and Bcl-3-p52 complexes [7]. Recently, a small zinc-binding B box domain within the core of USP domain, contributing to nuclear localization of CYLD, was identified [8]. However, the precise function of nuclear CYLD still remains elusive. Cytoplasmic CYLD acts as a negative regulator of NF-κB activation by deubiquitination of TNF receptor-associated factor (TRAF)-2/-6 and NF-κB essential modulator (NEMO) [9]. Little is known about the relevance of deubiquitinating enzymes for HCC development. We and others observed decreased levels of CYLD mRNA and protein in human HCC tissues [10], [11]. In addition, a negative correlation between CYLD and oncogenic c-MYC expression has been described [12]. In CYLD *knockout* mouse models, a central role of CYLD for liver injury and hepatocarcinogenesis was demonstrated. Liver-specific deletion of "full-length" CYLD, accompanied by expression of an ablated CYLD splice variant, resulted in chronic liver injury and profoundly increased sensitivity towards carcinogen-induced liver cancer [13]. Deletion of full-length CYLD resulted in spontaneous HCC development in another murine model [14]. Moreover, downregulation of CYLD was shown to contribute to apoptosis resistance of human HCC cells [11]. However, the exact role of CYLD in human HCC development and progression remains unclear.

In early stages HCC is curable by liver transplantation, surgical resection or local ablation [15], but most patients present with locally advanced and/or metastasized HCC at first diagnosis. Even after curative surgery or local ablation, long-term survival remains limited due to high incidence of intrahepatic recurrence. Identification of prognostic biomarkers would help to identify patients at high risk for recurrence. Here we show for the first time that lacking nuclear expression of the deubiquitinase CYLD in HCC tumor tissues strongly correlates with poor outcome of patients.

## Patients and Methods

### Patients' cohort and immunohistochemistry

All experiments were done in accordance with the governmental and institutional guidelines of the ICH-GCP (according to the principles expressed in the Declaration of Helsinki) and were performed under the written approval by the local ethics committee of the Medical Faculty of Heidelberg University (206/2005). For the evaluation of CYLD expression, a tissue microarray (TMA) was established including HCC samples of 95 patients, corresponding tumorous liver tissues and normal liver samples as previously described [16]. Briefly, representative tissue blocks were selected as donor blocks for the TMA. Sections were cut from each block and stained with hematoxylin and eosin. Representative regions were chosen from each of the HCC and normal liver tissue samples. One cylindrical core tissue specimens per tumor block (diameter 0.6 mm) was punched from these regions and arrayed into the recipient paraffin block using a semiautomatic system (Beecher Instruments, Silver Spring, Maryland, USA). TMA slides were dewaxed and rehydrated using xylene and a series of graded alcohols, followed by heat-induced antigen retrieval using citrate buffer (pH 6). Staining was performed with a NovoLink Min Polymer Detection System (Leica Microsystems, Wetzlar, Germany) according to the manufacturer's instructions. For CYLD detection a polyclonal rabbit anti-CYLD antibody (Abcam, ab137524), for Ki67 staining a monoclonal rabbit anti–Ki67 antibody (Bethyl Laboratories, Inc, Montgomery, USA, IHC-00375) was used. Unspecific binding was excluded by omitting the primary antibody. For the immunohistochemical, semiquantitative assessment of CYLD expression, the product of the scores of staining intensity and quantity of immunoreactive tumor cells was calculated as previously described [17]. Briefly, the final immunohistochemical score (IHS; ranging from 0 to 12) was obtained by multiplication of the intensity and the quantity score. To investigate the relationship between CYLD expression and survival, patients were grouped into two categories. Patients with a CYLD expression under the median expression value were assigned to the low-expression group. Patients with an expression level larger than or equal to the median value were assigned to the high-expression group. This procedure, which results in size-balanced patient groups, was independently applied to cytoplasmic and nuclear CYLD expression. For ^nuc^CYLD expression, levels ≥3, and for ^cyt^°CYLD expression, levels ≥6 were considered as high, respectively. Two pathologists independently assessed expression values. Sections were re-examined in case of disagreement.

All patients were treated at the University Hospital in Heidelberg, Germany, between 1998 and 2013. Decision for liver transplantation was made based on imaging findings in a multidisciplinary tumor board according to the national guidelines and the German Transplant Act, which are based on the Milan criteria and EASL guidelines [18]. The histological grade of tumor differentiation was assigned the Edmondson Grading System [19].

### Cell culture experiments

The immortalized liver cell line THLE-2, the hepatoblastoma cell line HepG2 and the HCC cell lines Huh7 and Hep3B were purchased from ECACC. Cells were cultured in DMEM (Invitrogen, Karlsruhe, Germany), supplemented with 10% fetal calf serum (FCS, Biochrom, Berlin, Germany), 1% Pen/Strep (PAA Laboratories, Pasching, Austria), 1% HEPES and 1% L-Glutamine (Cambrex, Verviers, Belgium). Cells were cultivated at 37°C with 5% CO_2_. Doxorubicin was purchased from Sigma-Aldrich (Hamburg, Germany).

For immunhistochemical staining 2.5×10^6^ cells were seeded in 12-well plates on cover glasses (18 mm diameter). After treatment, cells were fixed with 4% PFA and stained for CYLD (same procedure used for the TMA staining, dilution 1∶50, 1 h incubation). Hematoxylin and eosin (H&E) staining were performed according to standard procedures. Images were captured using an inverted microscope.

### Western blotting

Protein extraction and preparation of total, nuclear and cytoplasmic extracts were performed as previously described [20], [21]. Protein concentration was determined by Bradford protein assay (Bio-Rad, Munich, Germany). SDS-PAGE and Western blotting were performed according to standard procedures. Immunodetection was performed using the following primary antibodies: CYLD (E-4), IκB-α (C-21) (both Santa Cruz Biotechnology, Heidelberg, Germany), HDAC1 (Cell Signaling, Frankfurt, Germany) and α-Tubulin (Sigma-Aldrich, Munich, Germany).

### Statistical Analysis

Overall survival was calculated from the date of surgery (OS, event  =  death by any cause). Survival time was “censored” for patients who did not experience an event. Those parameters not available for the analysis are depicted as “NA” (not available). Recurrence free survival (RFS) was not included in the study due to limited availability of recurrence data.

The relationship between staining index score and patient's age and gender, Edmonson grading, tumor size, vascular infiltration, viral hepatitis, bilirubin, serum AFP and Child-Pugh score was tested using a Chi-square test and quantified by the Spearman's rank correlation coefficient. Cox regression models were applied to assess the influence of explanatory variables on overall survival. For each endpoint, explanatory variables with univariate (Cox regression) probability only values <0.1 were included into a multiple Cox regression model. Hazard ratios were calculated to quantify prognostic effects. The Kaplan-Meier method was used to depict overall survival rates and differences assessed by log-rank test. All analyses were performed using SPSS 20 (IBM, NY, USA). *P* values <0.05 were considered statistically significant.

## Results

### CYLD expression in human HCC tissues

**Figure 1:**
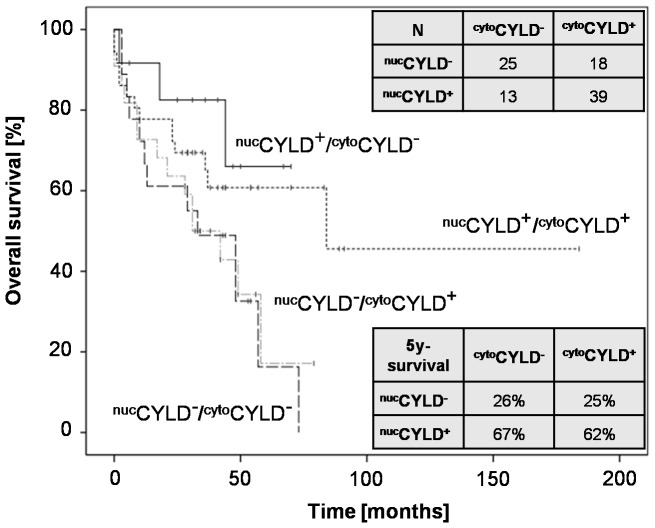
Survival of HCC patients after liver surgery according to subcellular CYLD expression. Kaplan-Meier analysis (N = 95) for overall survival (OS) of patients receiving liver resection, for the following subgroups: (^nuc^CYLD^+^/^cyt^°CYLD^−^, ^nuc^CYLD^+^/^cyt^°CYLD^+^, ^nuc^CYLD^−^/^cyt^°CYLD^+^ and ^nuc^CYLD^−^/^cyt^°CYLD^−^ (*P* = 0.06). Positive nuclear (^nuc^CYLD^+^) and cytoplasmic (^cyt^°CYLD^+^) CYLD staining was defined as an immunohistochemical score (IHS) ≥3 for ^nuc^CYLD^+^ and ≥6 for ^cyt^°CYLD^+^ (IHS ranging from 0 to 12, obtained by multiplication of the intensity and the quantity score).

**Figure 2:**
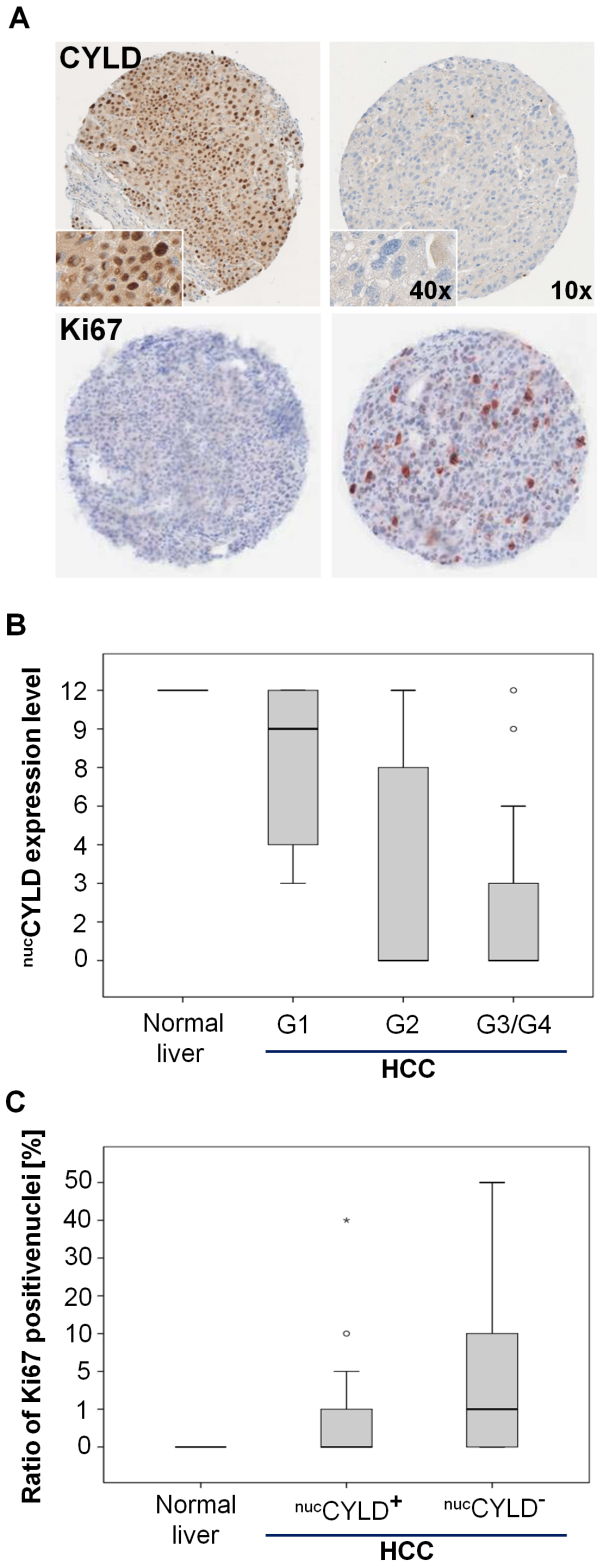
Nuclear CYLD expression in correlation to tumor grading in human HCC tissues. (**A**) Representative staining of positive and negative nuclear CYLD expression in HCC specimens. 10- and 40-fold magnification (^nuc^CYLD^+^; upper left and ^nuc^CYLD^−^, upper right panel). Representative staining of corresponding Ki67 expression in HCC specimens. 10-fold magnification (lower panels). (**B**) Nuclear expression of CYLD (IHS score 0–12) in HCC (G1 = 13, G2 = 54, G4 = 25 G4 = 3) and normal liver tissues (N = 7) correlated to grading (Spearman correlation coefficient: −0.423, P<0.001). (C) Nuclear expression of CYLD related to the ratio of Ki67 positive nuclei in HCC (^nuc^CYLD^+^; N = 47; ^nuc^CYLD^−^; N = 41) and normal liver tissues (N = 7) (Spearman correlation coefficient: −0.271, *P* = 0.006).

Downregulation of the deubiquitinase CYLD has been described as a frequent event in human HCC [10], [11]. In addition, CYLD deletion led to HCC development in murine *knockout* models [13], [14]. However, only little is known about subcellular CYLD localization and its association to the outcome of HCC patients. In order to assess CYLD expression, we analyzed 95 HCC tissue samples (N = 67 derived from liver resection, N = 28 from liver transplantation, respectively). Patients' characteristics concerning etiology, gender, and tumor stage are listed in Table 1. Subcellular CYLD expression was assessed (Fig. 1 and 2A, upper panels). 70 patients (74%) showed high nuclear and/or cytoplasmic CYLD expression, referred as ^total^CYLD^+^. In 25 patients (26%), only minor or absent CYLD expression was detected, both nuclear and cytoplasmic, referred as ^total^CYLD^−^. 52 patients (55%) showed positive nuclear staining for CYLD (^nuc^CYLD^+^). Among them, 39 (75%) were also positive for cytoplasmic expression (^nuc^CYLD^+^/^cyt^°CYLD^+^, Fig. 1). There was a positive correlation between ^nuc^CYLD^+^ and ^cyt^°CYLD^+^ (*P* = 0.001). To figure out differences in CYLD expression between patients treated with resection or transplantation, we compared subcellular CYLD expression in these two groups. CYLD expression levels for patients treated with resection or transplantation were similar (data not shown).

**Table 1:** Baseline characteristics of HCC patients corresponding to CYLD expression in the cytoplasma (^cyt^°CYLD^+/−^) or nucleus (^nuc^CYLD^+/−^).

Variables	N		^nuc^CYLD^−^		^nuc^CYLD^+^		*P* value	^cyt^°CYLD^−^		^cyt^°CYLD^+^		*P* value
**sex**	95		43		52		0.46	38		57		0.84
men	74	(78%)	35	(81%)	39	(75%)		30	(78%)	44	(77%)	
women	21	(22%)	8	(19%)	13	(25%)		8	(21%)	13	(23%)	
**grading**	95		43		52		0.038	38		57		0.55
G1	13	(14%)	3	(7%)	10	(19%)		5	(13%)	8	(14%)	
G2	54	(57%)	22	(51%)	32	(62%)		19	(50%)	35	(61%)	
G3	25	(26%)	17	(40%)	8	(15%)		13	(34%)	12	(21%)	
G4	3	(3%)	1	(2%)	2	(4%)		1	(3%)	2	(4%)	
**age**	95		43		52		0.11	38		57		0.93
<60 y	37	(39%)	13	(30%)	24	(46%)		15	(40%)	22	(39%)	
>60 y	58	(61%)	30	(70%)	28	(54%)		23	(60%)	35	(61%)	
**tumor size**	83		38		45		0.68	33		50		0.9
<5 cm	37	(45%)	16	(42%)	21	(47%)		15	(46%)	22	(44%)	
>5 cm	46	(55%)	22	(58%)	24	(53%)		18	(54%)	28	(56%)	
**vascular invasion**	83		37		46		0.83	33		50		0.01
negative	37	(45%)	16	(43%)	21	(46%)		9	(27%)	28	(56%)	
positive	46	(55%)	21	(57%)	25	(54%)		24	(73%)	22	(44%)	
**AFP**	81		36		45		0.065	32		49		0.02
<8 IU/ml	43	(53%)	15	(83%)	28	(62%)		12	(37,5%)	31	(63%)	
>8 IU/ml	38	(47%)	21	(17%)	17	(38%)		20	(62,5%)	18	(37%)	
**CHILD**	89		41		48		0.55	34		55		0.85
A	68	(77%)	33	(81%)	35	(73%)		25	(73%)	43	(78%)	
B	11	(12%)	5	(12%)	6	(12%)		5	(15%)	6	(11%)	
C	10	(11%)	3	(7%)	7	(15%)		4	(12%)	6	(11%)	
**alcohol consumption**	91		41		50		0.76	36		55		0.66
negative	68	(75%)	30	(73%)	38	(76%)		26	(72%)	42	(76%)	
positive	23	(25%)	11	(27%)	12	(24%)		10	(28%)	13	(24%)	
**viral hepatitis**	87		40		47		0.89	34		53		0.33
B	16	(18%)	7	(17,5%)	9	(20%)		8	(23,5%)	8	(15%)	
C	19	(22%)	8	(20%)	11	(23%)		9	(26,5%)	10	(19%)	
**bilirubin**	87		40		47		0.54	32		55		0.53
normal	58	(67%)	28	(70%)	30	(33%)		20	(63%)	38	(70%)	
increased	29	(33%)	12	(30%)	17	(67%)		12	(38%)	17	(30%)	

Bilirubin levels >1 mg/dl were depicted as “increased”. AFP serum levels: normal range <8 IU/ml).

### Correlation of CYLD with clinicopathological features

CYLD expression in HCC samples (both nuclear and cytoplasmic) was correlated with various clinicopathological features (Table 1). Median age of patients was 63 (±11.7) years (male∶female ratio 3.5∶1). 40% of patients had liver cirrhosis due to viral hepatitis B or C. Most patients had Child-Pugh score A (77%, B: 12%). No correlations between viral hepatitis or Child-Pugh score with ^nuc/cyt^°CYLD expression were observed. ^cyt^°CYLD^+^ had a significant correlation with vascular invasion (Table 1, *P* = 0.01) and serum AFP (Table 1, *P* = 0.02). In contrary, ^nuc^CYLD^+^ was not significantly associated with vascular invasion or serum AFP, but had a significant correlation with tumor grading (Table 1). Strikingly, the IHS for nuclear CYLD expression inversely correlated with both tumor grading (Spearman correlation coefficient −0.423, *P*<0.001, Fig. 2B) and Ki67-positivity (Spearman correlation coefficient −0.272, *P* = 0.005, Fig. 2C). In normal liver tissue (N = 7), median IHS for nuclear CYLD expression was 12. In HCC samples, the median score was 9 in G1 samples *vs.* 0 in G3/4 samples (Fig. 2B). 32% of the samples with a positive nuclear CYLD staining compared to 53% of negative samples showed a nuclear positivity of Ki67 defined as ≥1 Ki67-positive HCC cells per visual field, range 1–50 (data not shown).

### Correlation of CYLD expression with outcome

N = 95 patients were eligible for survival analysis. For patients undergoing HCC resection or liver transplantation the 1-/3-/5-year survival rates were 79/68/50% for patients with ^total^CYLD^+^, and 77/50/30% and for those with ^total^CYLD^−^, respectively (log-rank: *P* = 0.1, HR 1.6, data not shown).

In total, 3 variables were found to be significantly associated with overall survival (OS) in univariate Cox regression analysis (Table 2): a) a tumor diameter less than 5 cm (*P* = 0.007, HR = 0.4), b) AFP under 8 IU/ml (*P* = 0.015, HR 0.4) and c) negative nuclear CYLD expression (^nuc^CYLD^−^ = IHS≤3, *P* = 0.02, HR = 2.1). Strikingly, positive nuclear CYLD was a favourable prognostic factor for patients with HCC after surgery.

**Table 2:** Univariate Cox analysis of different clinical, laboratory and histopathological parameters and CYLD expression for overall survival (OS).

		Univariate					Univariate		
Variables (N)		N*	HR (95% CI)	Global *P* value	Variables (N)		N*	HR (95% CI)	Global *P* value
**sex**					**CHILD**				
men	(69)	35	0.98 (0.47–2.04)	0.95	A	(64)	27	0.55 (0.29–1)	0.07
women	(19)	9	(Ref.)		B/C	(20)	14	(Ref.)	
**grading**					**alcohol**				
1–2	(67)	27	0.55 (0.296–1.0)	0.05	negativ	(65)	34	1.87 (0.86–4.09)	0.12
3–4	(28)	17	(Ref.)		positive	(21)	8	(Ref.)	
**age**					**viral hepatitis**				
<60	(35)	16	0.66 (0.35–1.25)	0.2	negative	(49)	28	1.43 (0.738–2.75)	0.29
>60	(53)	28	(Ref.)		B/C	(34)	13	(Ref.)	
**tumor size**					**bilirubin**				
<5 cm	(37)	10	0.37 (0.18–0.76)	0.007	normal	(55)	26	0.93 (0.49–1.8)	0.84
>5 cm	(46)	27	(Ref.)		increased	(28)	14	(Ref.)	
**vascular invasion**					**^cyt^°CYLD**				
negativ	(37)	13	0.54 (0.27–1.08)	0.08	negative	(33)	17	1.16 (0.63–2.15)	0.63
positive	(46)	25	(Ref.)		positive	(55)	27	(Ref.)	
**serum AFP**					**^nuc^CYLD**				
<8 IU/ml	(42)	15	0.433 (0.22–0.85)	0.015	negative	(40)	27	2.14 (1.15–3.98)	0.02
>8 IU/ml	(35)	22	(Ref.)		positive	(48)	17	(Ref.)	

CI, confidence interval; HR, hazard ratio. N = 95; N*: Number of uncensored patients (patients who experienced the event).

**Figure 3:**
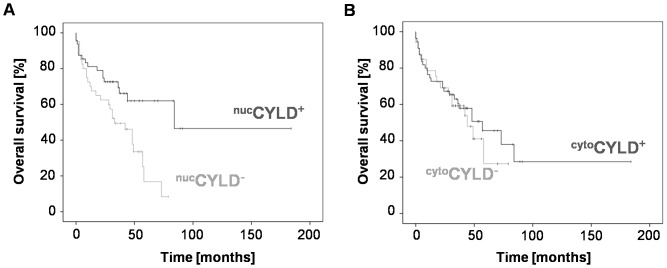
Overall survival of HCC patients with positive CYLD expression. (**A**) Overall Survival (OS) in patients with positive (^nuc^CYLD^+^; N = 48) and negative nuclear CYLD expression in HCC tissues (^nuc^CYLD^−^; N = 40; *P* = 0.007). (**B**) OS in patients with positive (^cyt^°CYLD^+^, N = 55) and negative cytoplasmic CYLD expression (^cyt^°CYLD^−^; N = 33; *P* = 0.6).

**Figure 4:**
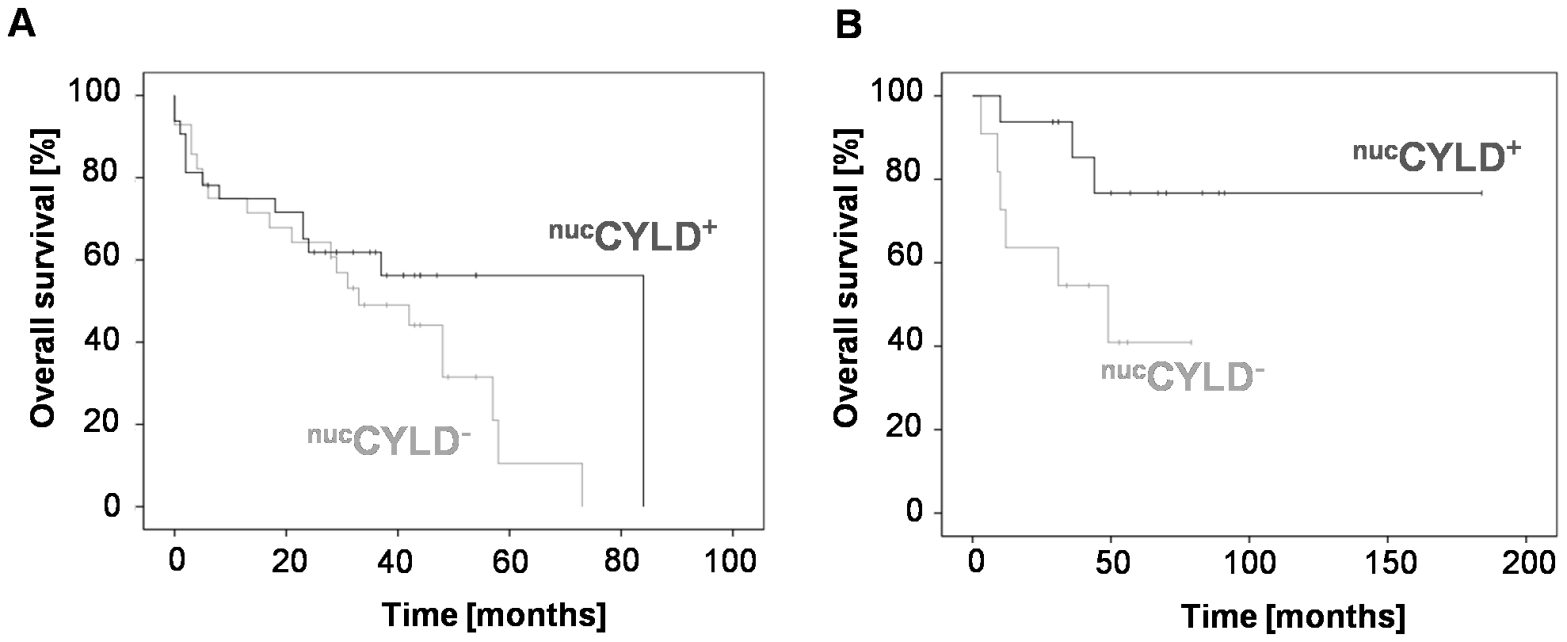
Correlation of CYLD expression with outcome after liver resection *vs.* liver transplantation. (**A**) Kaplan-Meier analysis for overall survival (OS) of patients receiving liver resection with positive (^nuc^CYLD^+^, N = 33) and negative nuclear CYLD expression (^nuc^CYLD^−^; N = 29; *P* = 0.1). (**B**) OS of patients receiving liver transplantation with positive (^nuc^CYLD^+^; N = 16) or negative nuclear CYLD expression (^nuc^CYLD^−^; N = 11; *P* = 0.04).

In the multiple Cox regression analysis, tumor size and CHILD score were independent prognostic factors (*P* = 0.01, HR = 0.34 and *P* = 0.04, HR = 0.4, Table 3). ^nuc^CYLD^+^ did not prove to be an independent prognostic parameter (*P* = 0.27, Table 3). ^cyt^°CYLD^−^ patients showed non-significant differences for overall survival compared to the ^cyt^°CYLD^+^ group (IHS<6, *P* = 0.6, Fig. 3B, HR = 1.2, respectively) Importantly, ^nuc^CYLD^+^ was significantly associated with improved OS in Kaplan-Meier analysis (^nuc^CYLD^+^: 1-/3-/5-year OS rates 81/73/63% *vs.* ^nuc^CYLD^−^: 75/50/26%, *P* = 0.007, Fig. 3A). The analysis was repeated with patients divided according to their surgical procedure (liver resection *vs.* liver transplantation). Patients in the ^nuc^CYLD^+^ group after liver resection showed a non-significant differences for OS, compared to the ^nuc^CYLD^−^ group (^nuc^CYLD^+^: 1-/3-/5-year survival rates 77/64/59% *vs.* ^nuc^CYLD^−^: 75/50/20%, *P* = 0.1, Fig. 4A). In patients after liver transplantation, ^nuc^CYLD^+^ was associated with longer OS compared to ^nuc^CYLD^−^, and a log-rank test confirmed significance (^nuc^CYLD^+^: 1-/3-/5-year survival rates 94/94/77% *vs.* ^nuc^CYLD: 73/55/41%, *P* = 0.04, Fig. 4B). Analyzing CYLD expression in all subgroups (^nuc^CYLD^+^/^cyt^°CYLD^−^, ^nuc^CYLD^+^/^cyt^°CYLD^+^, ^nuc^CYLD^−^/^cyt^°CYLD^+^ and ^nuc^CYLD^−^/^cyt^°CYLD^−^), no significant differences for overall survival were detected by the Kaplan-Meier approach. Among the subgroups, ^nuc^CYLD^+^/^cyt^°CYLD^−^ had the most favourable 5-year survival rate (*P* = 0.06; Fig.1).

**Table 3:** Multivariate Cox regression analysis of CLYD expression and clinical/laboratory/histopathological features for the prediction of overall survival (OS).

	Multivariate			
Variables	(N)	N*	HR (95% CI)	Global *P* value
**CHILD**				
A	(53)	21	0.4 (0167–0.97)	0.04
B/C	(15)	9	(Ref.)	
**grading**				
1–2	(47)	17	0.7 (0.296–1.698)	0.44
3–4	(21)	13	(Ref.)	
**^nuc^CYLD**				
negative	(27)	16	1.56 (0.71–3.41)	0.27
positive	(41)	14	(Ref.)	
**tumor size**				
<5 cm	(32)	8	0.34 (0.14–0.797)	0.01
>5 cm	(36)	22	(Ref.)	
**vascular invasion**				
negative	(29)	9	1 (0.392–2.68)	0.96
Positive	(39)	21	(Ref.)	
**serum AFP**				
<8 IU/ml	(38)	12	0.4 (0.17–1)	0.07
>8 IU/ml	(30)	18	(Ref.)	

CI, confidence interval; HR, hazard ratio. N = 95; N*: Number of uncensored patients (patients who experienced the event).

### Subcellular CYLD expression in HCC cell lines

To assess subcellular CYLD expression *in vitro*, total, cytoplasmic and nuclear CYLD expression levels in the non-malignant liver cell line THLE-2, the HCC cell lines Hep3B, Huh7 and in the hepatoblastoma cell line HepG2 were analyzed. Total and cytoplasmic CYLD expression was low in all 3 malignant cell lines compared to THLE-2 cells. Nuclear CYLD expression levels were hardly detectable, with no differences between the non-malignant liver cell line and liver cancer cells (Figure S1).

We have already shown that *knockdown* of CYLD increased the resistance towards chemotherapy [22]. Here, we observed induction of CYLD expression after chemotherapy with doxorubicin, which is frequently used for transarterial chemoembolization in HCC patients. The following analysis of subcellular CYLD expression 6 and 12 h after doxorubicin treatment revealed increased total, cytoplasmic and nuclear CYLD expression levels. This indicates a predominate role of increased CYLD protein translation rather than enhanced nuclear shuttling (Figure S2A, B). We additionally analyzed the TMA data and correlated cytoplasmic and nuclear CYLD expression. In cases with a cut-off value>3, ^nuc^CYLD was also increased (Figure S2C). Immunohistological CYLD staining of HepG2 and Huh7 cells showed increased CYLD levels after doxorubicin treatment and additionally revealed induction of CYLD expression in nucleoli (Figure S3).

## Discussion

In the present study, the clinicopathological relevance of the deubiquitinase CYLD was assessed in a series of 95 human hepatocellular carcinoma (HCC) samples, obtained from patients undergoing resection or liver transplantation as treatment of early stage HCC. Our key finding is a strong correlation of nuclear CYLD expression with overall survival of HCC patients. Importantly, nuclear CYLD inversely correlated with both tumor grading and Ki67 positivity in HCC patients.

Originally, CYLD has been described as a tumor suppressor gene in a rare tumor disease, called familial cylindromatosis (Brooke-Spiegler Syndrome) [4]. In recent studies, downregulation of CYLD has been described in various other malignancies, including HCC [10], [11]. A reduced copy number of the CYLD gene was observed in more than 30% of human HCCs caused by chronic hepatitis C [23].

Suppression of CYLD is known to trigger oncogenic signaling, such as NF-κB, Wnt/ß-catenin and Bcl-3. However, the exact role of CYLD in cancer development is not fully understood. In murine *knockout* models, we and others revealed a central role of CYLD for hepatocarcinogenesis [12], [13], [14]. Activation of NF-κB signaling and chronic inflammation are discussed to trigger HCC development in mice lacking hepatic CYLD expression [9], [10].

CYLD has been shown to be regulated at both transcriptional and post transcriptional level [24]. This opens up opportunities for targeting CYLD expression in human HCC. Constitutive activation of the Ras-Raf-MEK-ERK (MAPK; mitogen-activated protein kinase) signaling pathway is the major upstream signaling mechanism responsible for constitutive expression of the CYLD transcription inhibitor Snail1 [5]. We have already shown that CYLD expression in HCC cells can be triggered by the multikinase inhibitor sorafenib, by inhibition of Raf-1, as well as by blockage of the pro-survival kinases MEK and EGFR and identified the recovery of CYLD expression as an interesting approach for overcoming HCC resistance [22].

So far, the pathophysiological role of subcellular CYLD localization remains elusive. Various deubiqitinating enzymes are predominantly localized in the nucleus. Nuclear DUBs regulate major cellular processes such as gene expression, DNA repair and DNA replication. Deubiquitination of chromatin substrates impacts these processes at several levels, directly by altering protein stability (K-48) and localization (K-63-linked polyubiquitination) [6]. Nuclear USP7 has been shown to deubiquitinate forkhead box-containing transcription factor 4, provoking its nuclear export and hence its inactivation [25]. However, CYLD has been described to be mainly located in the cytoplasma and the perinuclear region [26], [27]. Our study illustrates reduced nuclear CYLD expression in liver cancer cell lines. A correlation of CYLD mRNA and protein expression was described in previous studies [10]. Doxorubicin, which is a common agent used for transarterial chemoembolization procedures in HCC, induced CYLD expression in various subcellular compartments, including nucleoli. In addition, subcellular CYLD localization might be influenced by genetic alterations. In HEK293 cells, mutated, but not wildtype CYLD appeared in the nucleus. Overexpression of CYLD carrying a mutation of the small zinc-binding B box domain within the core of CYLDs USP domain led to nuclear accumulation of CYLD. Mutated CYLD showed equal DUB activity and specificity [8]. Our study showed that CYLD is able to locate in the nucleus. Further studies are required to elucidate the precise role of the b box domain and to uncover the molecular functions of nuclear CYLD. In HCC, it is still not known how subcellular CYLD localization is regulated.

This is the first study to present nuclear CYLD expression and its correlation with the outcome of HCC patients. Recently, it was reported that low CYLD mRNA and protein expression was paralleled by poor survival. In 76% of the HCC cases (N = 70, all patients undergoing resection) positive CYLD protein expression was described [28]. In our study, 74% out of 95 patients had positive nuclear and/or cytoplasmic expression. 41% out of 95 patients were positive for both nuclear and cytoplasmic CYLD expression (^cyto/nuc^CYLD^+^). We observed a significant association of ^cyt^°CYLD and ^nuc^CYLD positivity. However, 25% of HCC tissues with positive nuclear CYLD expression were lacking cytoplasmic expression. Interestingly, a lack of nuclear CYLD expression correlated with poor differentiation (evaluated by Edmonson grading) and higher Ki67 positivity. It is well known that increased proliferation, indicated by high levels of Ki67 positive cells, is associated with earlier HCC recurrence after resection [29], [30], [31]. In addition, poor differentiation is associated with vascular invasion and unfavourable prognosis [32], [33]. It is not known, however, whether increased proliferation or poor differentiation might influence nuclear CYLD expression or *vice versa*. An inverse correlation of CYLD protein expression with mitotic activity (as assessed by the MIB1-index), but not with tumor stage, has been reported by others in human HCC [12].

Resection of HCC remains the mainstay of treatment in patients with limited tumor size, normal bilirubin levels and the absence of portal hypertension [34]. However, liver transplantation is widely accepted as a curative therapeutic approach in well-selected patients with early stage HCC [35]. After liver transplantation, the 5-year survival rate was 63% in our study. Similar survival data after liver transplantation have been reported by other centers [36], [37]. Following tumor resection 5-year survival rate was 31%. Other known well-defined factors, such as tumor size and Child-Pugh score, were confirmed as independent prognostic factors in patients after HCC resection or liver transplantation in our study [38]. The inferior survival rates after resection compared to transplantation, underline the clinical benefit of transplantation in selected patients [39].

Importantly, positive nuclear CYLD expression was significantly associated with improved OS in the cohort of resected and transplanted patients. So far, adjuvant treatment approaches are not established after curative surgery of HCC. Since the present retrospective study identifies a subgroup of patients with improved OS after curative resection and liver transplantation (^nuc^CYLD^+^), nuclear CYLD expression might be an interesting biomarker for prospective clinical trials in the adjuvant setting after HCC surgery.

So far, the functional role of nuclear CYLD remains unclear. We cannot exclude that nuclear expression of CYLD might be an early event in HCC development, which disappears upon tumor progression, or CYLD simply changes its subcellular location. Further insights into the regulation of subcellular CYLD expression would help to evaluate the pathophysiological contribution of CYLD localization to hepatocarcinogenesis and HCC progression.

In conclusion, we identified nuclear CYLD expression as a prognostic parameter for patients undergoing surgery in HCC patients. Thus, nuclear CYLD is a promising novel marker to predict outcome of HCC patients, and might help to identify patients with a reduced risk of recurrence after resection or transplantation.

## Supporting Information

Figure S1 **Low CYLD expression in HCC cell lines.** Western blot analysis of basal CYLD expression levels in total, cytoplasmic and nuclear cell extracts derived from THLE-2 (non-malignant liver cell line), HepG2, Huh7 and Hep3B cells. Tubulin served as a loading control for total and cytoplasmic fractions, HDAC1 for nuclear fractions.
(TIF)

Figure S2 **Subcellular CYLD expression in HCC cells after doxorubicin treatment and in TMA specimens.** (**A**) HepG2 and (**B**) Huh7 cells were treated with doxorubicin for 6 and 12 h (1 µM). Western blot analysis of CYLD expression in total, cytoplasmic and nuclear cell extracts (upper panel). Analysis of CYLD expression in total, cytoplasmic and nuclear cell extracts from HCC cells 12 h after doxorubicin treatment on the same gel (lower panel). Tubulin served as loading control for total and cytoplasmic fractions, HDAC1 for nuclear fractions. (**C**) Boxplot summarizing nuclear expression of CYLD (IHS 0–12) within categories of cytoplasmic expression (IHS 0–12) in HCC patients.
(TIF)

Figure S3 **Immunohistochemical CYLD staining of HCC cells.** Representative pictures of H&E and CYLD staining of untreated and doxorubicin treated HepG2 (left panels) and Huh7 cells (right panels). Magnification 100x, scale bar 30 µm. Arrows indicate nucleoli.
(TIF)
