# Supplementary figures and images for: Nuclear Expression of the Deubiquitinase CYLD Is Associated with Improved Survival in Human Hepatocellular Carcinoma
Source: PLoS One. 2014 Oct 16;9(10):e110591. doi: 10.1371/journal.pone.0110591 (PMC4199737; doi:10.1371/journal.pone.0110591)

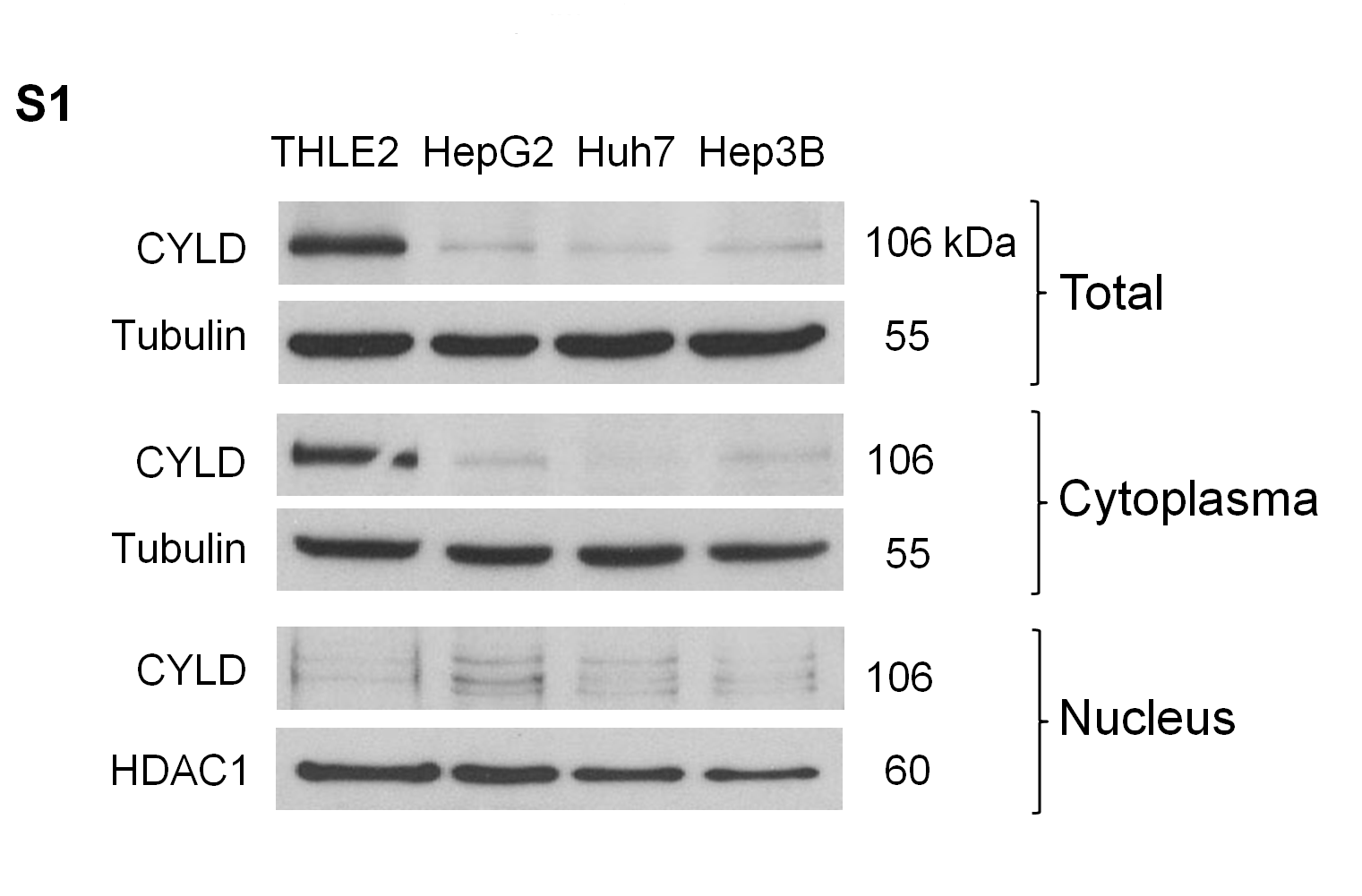

Supplement: Figure S1 — Low CYLD expression in HCC cell lines. Western blot analysis of basal CYLD expression levels in total, cytoplasmic and nuclear cell extracts derived from THLE-2 (non-malignant liver cell line), HepG2, Huh7 and Hep3B cells. Tubulin served as a loading control for total and cytoplasmic fractions, HDAC1 for nuclear fractions. (TIF) [file pone.0110591.s001.tif]

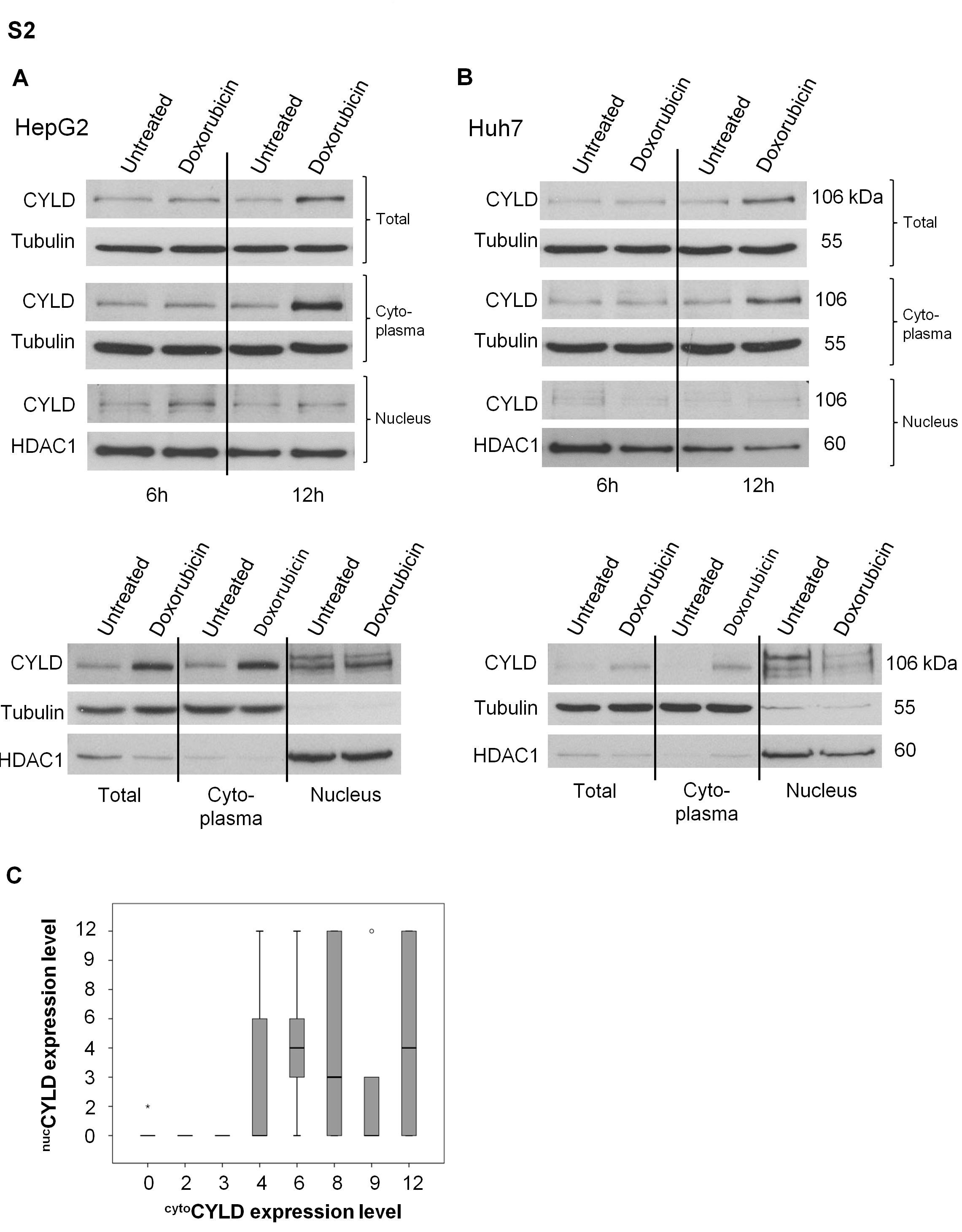

Supplement: Figure S2 — Subcellular CYLD expression in HCC cells after doxorubicin treatment and in TMA specimens. (A) HepG2 and (B) Huh7 cells were treated with doxorubicin for 6 and 12 h (1 µM). Western blot analysis of CYLD expression in total, cytoplasmic and nuclear cell extracts (upper panel). Analysis of CYLD expression in total, cytoplasmic and nuclear cell extracts from HCC cells 12 h after doxorubicin treatment on the same gel (lower panel). Tubulin served as loading control for total and cytoplasmic fractions, HDAC1 for nuclear fractions. (C) Boxplot summarizing nuclear expression of CYLD (IHS 0–12) within categories of cytoplasmic expression (IHS 0–12) in HCC patients. (TIF) [file pone.0110591.s002.tif]

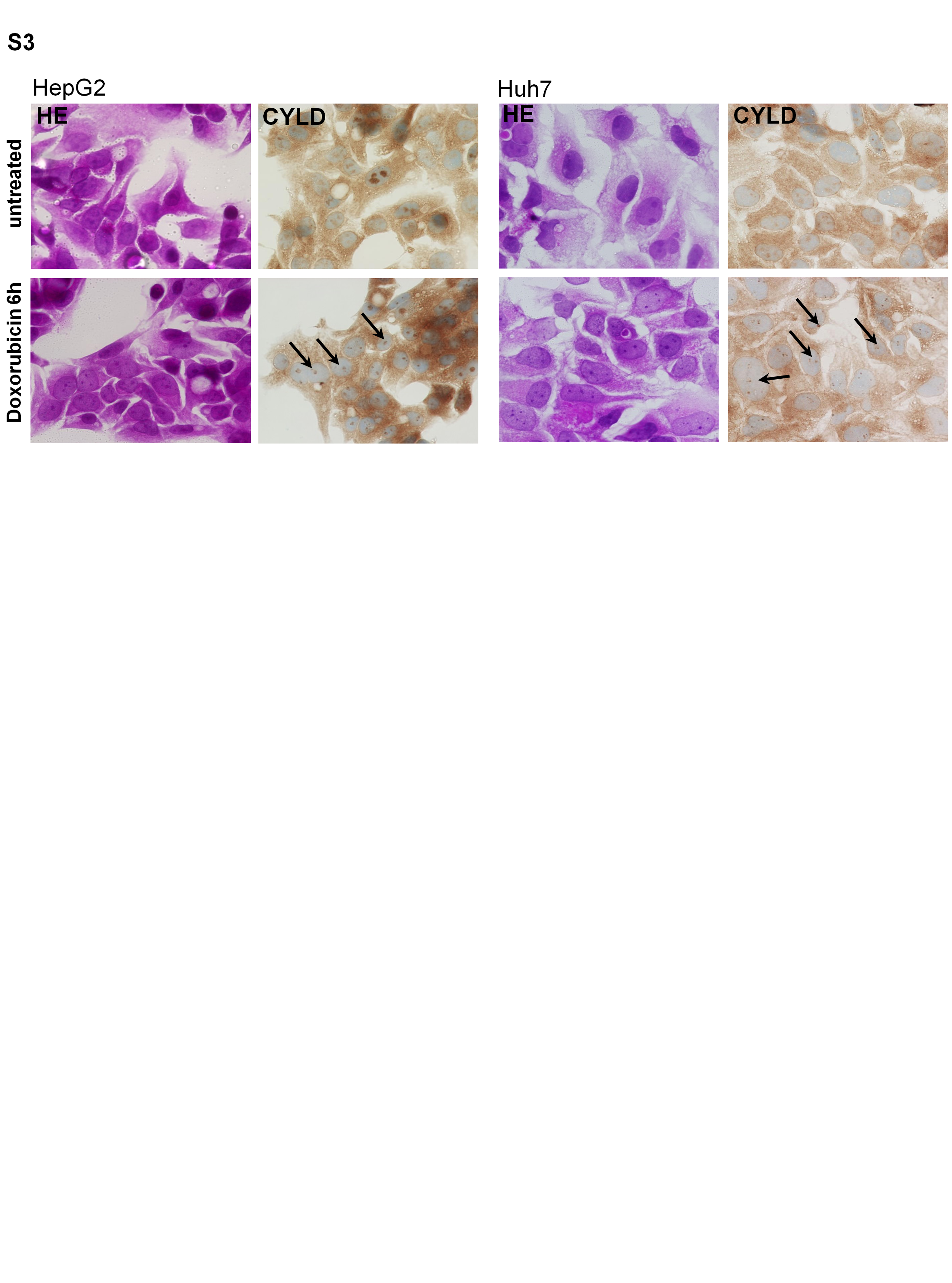

Supplement: Figure S3 — Immunohistochemical CYLD staining of HCC cells. Representative pictures of H&E and CYLD staining of untreated and doxorubicin treated HepG2 (left panels) and Huh7 cells (right panels). Magnification 100x, scale bar 30 µm. Arrows indicate nucleoli. (TIF) [file pone.0110591.s003.tif]
